# Platelets confound the measurement of extracellular miRNA in archived plasma

**DOI:** 10.1038/srep32651

**Published:** 2016-09-13

**Authors:** Adam J. Mitchell, Warren D. Gray, Salim S. Hayek, Yi-An Ko, Sheena Thomas, Kim Rooney, Mosaab Awad, John D. Roback, Arshed Quyyumi, Charles D. Searles

**Affiliations:** 1 Division of Cardiology, Department of Medicine, Emory University, Atlanta, GA, USA; 2 Department of Biostatistics, Rollins School of Public Health, Emory University, Atlanta, GA, USA.; 3 Department of Pathology, Emory University, Atlanta, GA, USA; 4 Section of Cardiology, Atlanta VA Medical Center, Decatur, GA, USA

## Abstract

Extracellular miRNAs are detectable in biofluids and represent a novel class of disease biomarker. Although many studies have utilized archived plasma for miRNA biomarker discovery, the effects of processing and storage have not been rigorously studied. Previous reports have suggested plasma samples are commonly contaminated by platelets, significantly confounding the measurement of extracellular miRNA, which was thought to be easily addressed by additional post-thaw plasma processing. In a case-control study of archived plasma, we noted a significant correlation between miRNA levels and platelet counts despite post-thaw processing. We thus examined the effects of a single freeze/thaw cycle on microparticles (MPs) and miRNA levels, and show that a single freeze/thaw cycle of plasma dramatically increases the number of platelet-derived MPs, contaminates the extracellular miRNA pool, and profoundly affects the levels of miRNAs detected. The measurement of extracellular miRNAs in archived samples is critically dependent on the removal of residual platelets prior to freezing plasma samples. Many previous clinical studies of extracellular miRNA in archived plasma should be interpreted with caution and future studies should avoid the effects of platelet contamination.

Extracellular miRNAs have been detected in various biofluids and are now considered to be biomarkers of disease. Although numerous high impact and carefully designed studies have identified extracellular miRNA profiles associated with different disease states, including cancer, neurologic disease, rheumatic disease and cardiovascular disease, clinical application of extracellular miRNAs as biomarkers has been hampered by inconsistencies in the literature. These inconsistencies are likely largely due to methodological variations in measurement of extracellular miRNA. The value of extracellular miRNAs as clinical biomarkers is critically dependent on identifying factors responsible for inter-study variability and the development of standardized protocols.

Repositories of human tissues and biofluids have been essential resources for biomarker discovery, and archived plasma has been utilized in many extracellular miRNA studies^1^,^2^,^3^(Table 1). It has been recognized that the presence of residual cells - particularly platelets - in plasma samples can influence miRNA assessment. Current recommendations call for platelet poor plasma (PPP), which is generated by centrifuging whole blood (collected in EDTA or sodium citrate vacutainers) twice prior to freezing - the first centrifugation to remove the bulk of circulating cells, the second to remove residual platelets. However, for many studies, the details of plasma storage and residual platelet removal were either not performed or not reported^4^,^5^,^6^(Table 1). Here, we studied archived human plasma samples from an established repository for the purpose of identifying extracellular miRNAs associated with peripheral artery disease (PAD). A striking degree of correlation between miRNA levels was observed among all samples, and study subjects’ platelet counts at the time of blood draw correlated significantly with plasma miRNA concentrations. Despite adhering to a recommended protocol for removing residual platelets and generating platelet-poor plasma from archived samples^7^, we suspected contamination by platelet miRNAs. We demonstrate that, for archived plasma samples containing residual platelets, a single freeze/thaw cycle results in the release of substantial quantities of miRNA and platelet microparticles (PMPs), thereby contaminating the extracellular pool of miRNA. These data reinforce the critical importance of plasma processing in extracellular miRNA studies and suggest that archived plasma samples not correctly processed prior to storage - the case for a large number of studies - are confounded by platelet miRNA contamination.

## Results

### Peripheral artery disease (PAD) case-control cohort characteristics

Cases were matched to controls with respect to the majority of cardiovascular disease (CVD) risk factors, however there were differences in the gender distribution, high density lipoprotein (HDL), and creatinine levels of cases compared to controls (Supplemental Table 1).

### miRNA levels are highly correlated and positively associated with platelet count

Five plasma miRNAs that were among the most differentially expressed between cases and controls in the discovery phase (miR-17-5p, -19a-5p, -19b-5p, -26b-5p, and -93-5p) were selected for further measurement in an additional 25 cases and 27 controls. In addition, 5 candidate miRNAs previously implicated in vascular disease (miR-21-5p, -126-3p, -126-5p, -145-5p, -222-5p) were measured in the validation cohort. In total, 12 plasma miRNAs (including Cel-39 and miR-425) were quantified in 52 samples. One sample was excluded from the final analysis due to poor overall PCR amplification. Measurement of spiked-in exogenous Cel-39 demonstrated low technical variability between samples.

**Figure 1:**
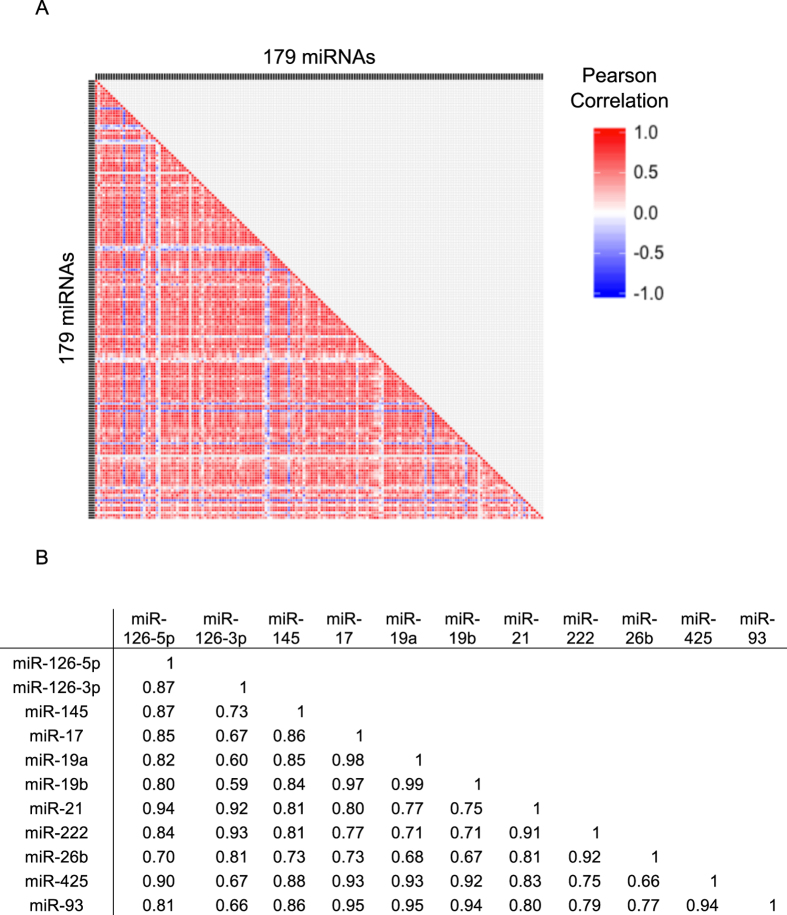
Correlation between miRNA levels in a case-control study of PAD utilizing archived plasma. Archived plasma samples were thawed and underwent additional centrifugation (1,900x g, 10 minutes) prior to RNA isolation and RT-qPCR. Data was normalized to the spike-in control. (**A**) Pearson correlation coefficients calculated between each pair of 179 miRNAs in the discovery cohort displayed a high degree of correlation between most miRNAs. (**B**) The 11 miRNAs measured in the validation cohort displayed very strong correlations between each other and all are statistically significant (P < 0.05).

**Figure 2:**
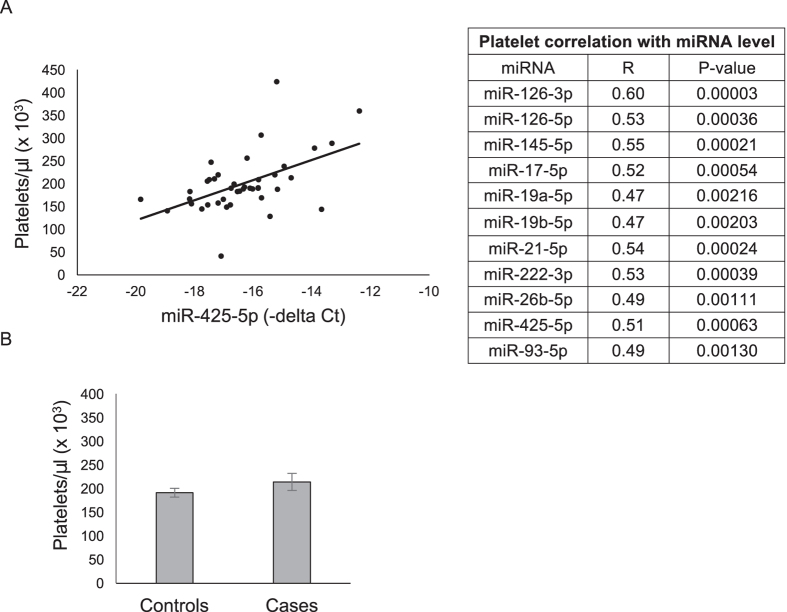
Correlation between miRNA levels and baseline platelet count in a case-control study of PAD utilizing archived plasma. (**A**) Among 41 samples for which platelet count was available, a significant positive correlation was observed between platelet count and miRNA level. The relationship between platelet count and miR-425 is depicted (left), and the correlation coefficient (R) and P-value for miRNAs measured in the validation cohort can be found in the table (right). (**B**) Platelet count did not differ between cases and controls (bottom).

A strikingly high degree of correlation was observed among almost all miRNAs measured in the discovery phase (Fig. 1A) and among all miRNAs measured in the validation phase (Fig. 1B), suggesting a common cellular origin. Study participants’ platelet counts, obtained by standard complete blood cell (CBC) analysis and performed at the time of blood draw, positively correlated with miRNA level (R = 0.51, P = 0.0006) (Fig. 2A). Platelet counts did not significantly differ between cases and controls (214 × 10^3^ platelets/μl ± 18 versus 191 × 10^3^ platelets/μl ± 9, respectively; p = 0.21) (Fig. 2B).

### Residual platelets in standard plasma preparations confound MP and miRNA detection after freeze/thaw, even after post-thaw processing

**Figure 3:**
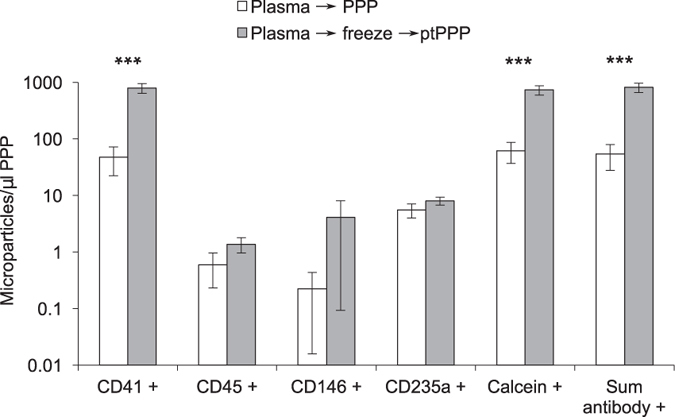
Freeze/thaw of standard plasma increased platelet MPs in platelet poor plasma (PPP). Standard plasma from six healthy volunteers was either immediately processed to PPP or exposed to a single freeze/thaw cycle prior to a second centrifugation step to remove residual platelets. Platelet (CD41+), leukocyte (CD45+), endothelial (CD146+), and RBC (CD235a) MPs were quantified by flow cytometry. Total number of MPs in a sample was either calculated by the sum of positive events detected by antibody staining (Sum antibody+) or measured by staining with calcein-AM, a non-specific dye that fluorescently labels intact vesicles. Results were similar and showed marked increase after freeze-thaw of standard plasma ^∗∗∗^indicates P < 0.001. This effect was largely due to an increase in platelet derived MPs (CD41+).

**Figure 4:**
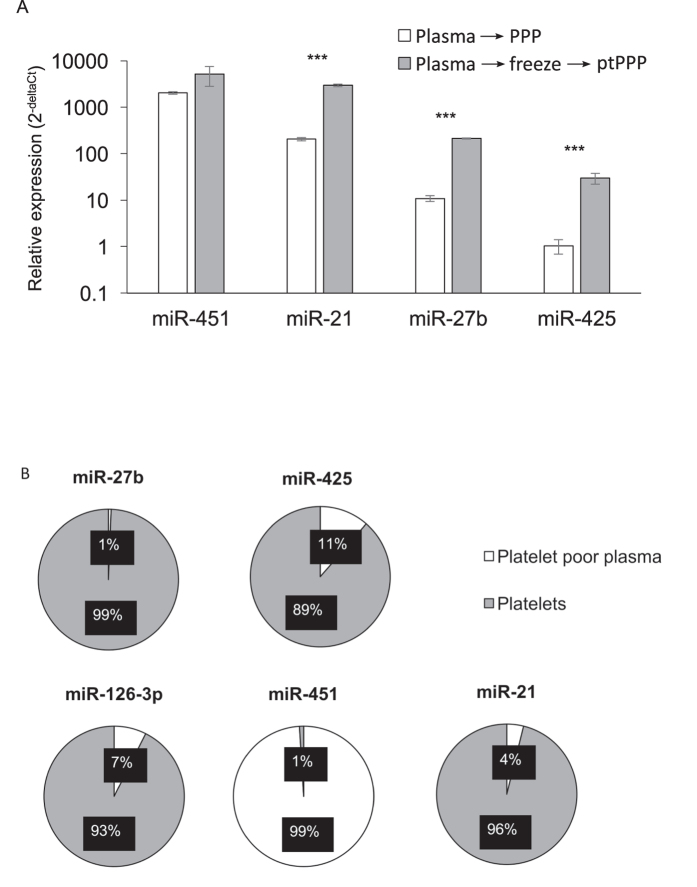
Freeze/thaw of standard plasma increased miRNA abundance in platelet poor plasma (PPP). (**A**) Select miRNAs were measured in PPP with or without an intermediate freeze/thaw cycle. miR-21, -27b, and -425 were markedly elevated after freeze/thaw, while the red blood cell specific miR-451 was not significantly different. (**B**) The platelet contribution of select miRNAs to standard plasma was calculated by subtracting the normalized relative expression (2^−(Ct miR-Ct Cel-39)^) in PPP from the relative expression in standard plasma and expressed as a fraction of the whole. With the exception of miR-451, these miRNAs are expressed at much greater levels in platelets than in the extracellular environment.

Utilizing blood samples obtained from healthy volunteers, we tested the assertion that post-thaw processing of archived plasma (to generate post-thawed PPP, or “ptPPP”) could effectively eliminate platelet contamination, as was previously suggested^7^. Protocols for the preparation of standard plasma from EDTA-anticoagulated blood typically call for centrifugation at 1000–2000x g for 10 minutes. However, the resultant plasma is well known to contain residual platelets. Here, ptPPP prepared after a single freeze/thaw cycle of standard plasma (EDTA-anticoagulated blood) had an approximately 20-fold increase in the number of platelet (CD41+) MPs compared to PPP prepared without freezing (798 ± 153 vs. 47 ± 25; P = 0.0007) (Fig. 3). Freeze/thaw did not significantly affect the levels of MPs from other sources (i.e. RBCs, leukocytes, endothelial cells). To assess whether freeze/thaw resulted in increased levels of miRNA detected in ptPPP, four miRNAs, (miR-21, -27b, -425, and -451) were measured in PPP and ptPPP, prepared after one freeze/thaw cycle. There were dramatic increases in miR-21, -27b, and -425 detected in ptPPP compared to PPP (Fig. 4). As expected, levels of miR-451, a RBC-specific miRNA not known to be appreciably expressed in platelets, were not significantly altered by freeze/thaw. In a separate experiment, the contribution of residual platelets to miRNA levels in fresh standard plasma (from EDTA-anticoagulated blood) was calculated for several miRNAs by subtracting the relative expression (2^−(Ct miR-Ct Cel-39)^) in PPP from the relative expression in fresh standard plasma (Fig. 4). Residual platelets potentially contribute 89–99% to levels of miR-21, miR-27b, miR-126, and miR-425 in standard plasma, whereas their contribution to the level of RBC-specific miR-451 is 1%. These data suggest that freeze/thaw of standard plasma, without removing residual platelets prior to freezing, results in a dramatic alteration of the MP and extracellular miRNA content of plasma, even after further post-thaw attempts to remove residual platelets.

### Residual platelets in standard plasma generate MPs and lose membrane integrity after freeze/thaw

**Figure 5:**
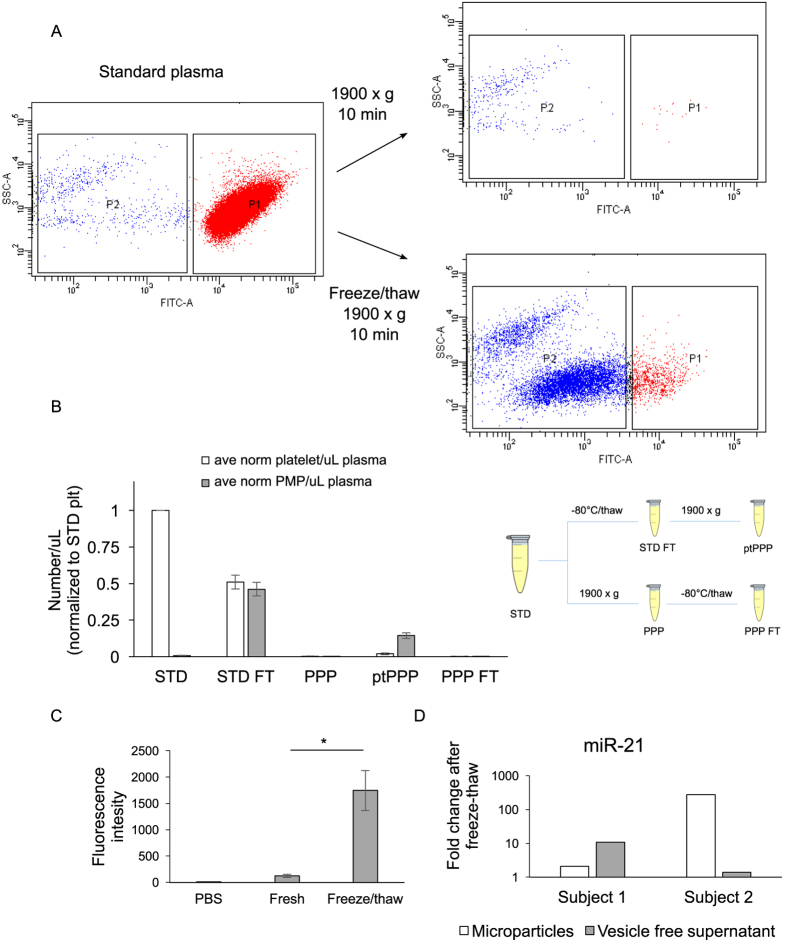
Freeze/thaw of residual platelets results in loss of membrane integrity, the formation of MPs, and the release of intracellular contents. (**A**) Representative scatterplots of standard plasma and PPP prepared without (top right) or with (bottom right) a freeze/thaw cycle. The X-axis is FITC (calcein intensity) and the Y-axis is side-scatter (SSC). The platelet gate is labeled as P1 (red) and the MP gate is labeled as P2 (blue). (**B**) Platelets and platelet-derived MPs (PMP) were measured in plasma samples from three healthy volunteers prepared as depicted in the cartoon: STD (standard plasma); STD FT (standard plasma, after freeze/thaw); PPP (platelet-poor plasma, after second centrifugation - 1900x g, 10 minutes – of standard plasma); FT PPP (platelet poor plasma, after second centrifugation of freezethaw standard plasma); PPP FT (freshly prepared PPP, then freeze/thaw). (**C**) Platelets were isolated from standard plasma, re-suspended in PBS, and stained with Calcein-AM. Platelets were either kept at room temperature or exposed to a single freeze/thaw cycle at −80 °C before centrifugation to sediment cells and MPs. Calcein that had escaped platelets, indicating loss of membrane integrity, was measured in the supernatant and was significantly higher in the supernatant from platelets exposed to freeze/thaw. (**D**) Freshly prepared plasma from 2 healthy subjects was prepared +/− a freeze/thaw cycle and miR-21 levels were measured in subsequently isolated MPs or the vesicle free supernatant.

EDTA-anticoagulated blood samples were obtained from three healthy volunteers and further processed as: standard plasma (STD); freeze/thaw standard plasma (STD FT); PPP; ptPPP; and PPP freeze/thaw (PPP FT = preparation of fresh PPP, followed by freeze/thaw). Platelets (CD41+, calcein++) and platelet-derived MPs (CD41+, calcein+) were measured in each preparation. A large number of platelet MPs were generated in STD FT, resulting in an approximately 20-fold increase in the number of MPs detected in ptPPP (STD FT vs ptPPP, Fig. 5A,B).

We asked whether freeze/thaw of residual platelets contaminates extracellular miRNA levels through the generation of MP-encapsulated miRNAs and/or non-encapsulated miRNAs into the plasma, so we first examined the effects of freezing/thawing on platelet membrane integrity. Residual platelets were isolated from STD and stained with calcein-AM, a membrane-impermeant fluorophore when activated. Freeze/thaw of these platelets resulted in a loss of membrane integrity as indicated by increased levels of calcein-AM in the supernatant after centrifugation (16,100x g, 20 minutes) (Fig. 5C), suggesting that cytosolic contents, including non-encapsulated miRNAs, were released. As expected, we found elevated levels of miR-21 in both the MP- and vesicle-free fractions of ptPPP samples (Fig. 5D). Interestingly, the relative abundance of miR-21 in the MP and vesicle-free fractions of ptPPP was different between the two blood samples; one sample had a 10-fold increase in miR-21 in the vesicle-free fraction and a relatively small increase in the MP fraction, while the other had an almost 300-fold increase in miR-21 in the MP fraction and a relatively small increase in the vesicle free fraction (Fig. 5A). This finding suggests that miRNAs from residual platelets contaminate the post-thaw extracellular miRNA pool through multiple mechanisms that vary from sample to sample.

### Effect of freeze/thaw on miRNA levels in EDTA versus citrate anticoagulated blood

**Figure 6:**
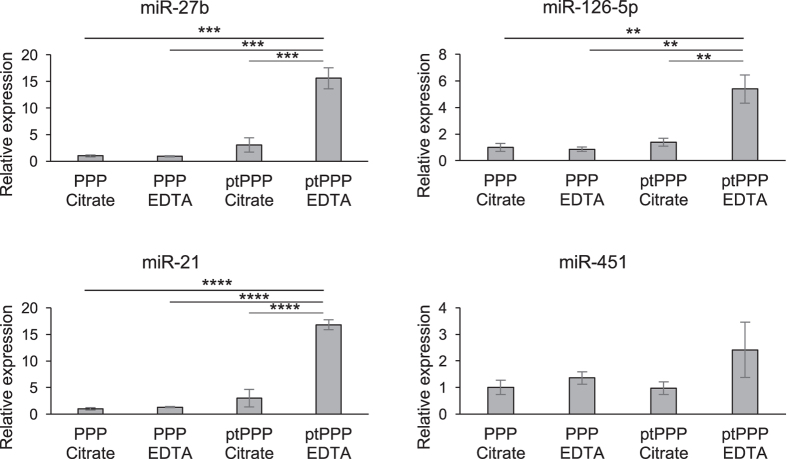
The effect of freeze/thaw on PPP isolated from EDTA- or citrate-anticoagulated blood. Standard plasma from 3 healthy volunteers was separated from EDTA- or citrate-anticoagulated blood (3,000 RPM/1,800x g; 10 minutes). EDTA tubes contained 10 mL of whole blood while citrate tubes contained 2.7 mL. Standard plasma was either exposed to a freeze/thaw cycle before a second centrifugation or processed without freezing, and select miRNAs were measured in the resulting PPP. Treatment groups were compared using a one-way ANOVA with a Tukey’s post-hoc test for multiple comparisons (** indicates P < 0.01, ***P < 0.001, ****P < 0.0001).

Blood was collected from three healthy volunteers into EDTA and sodium citrate vacutainers, and the effect of freeze/thaw on levels of select miRNAs in plasma was assessed. Whereas there were no differences in miRNA levels between fresh citrate and EDTA PPP, freeze/thaw dramatically increased of levels of platelet miRNAs (miR-27b, -21, and -126-5p) in EDTA ptPPP compared to citrate ptPPP. In contrast, levels of RBC miR-451 were not significantly different between EDTA and citrate ptPPP (Fig. 6).

In comparing MP profiles between citrate and EDTA PPP, we also observed substantially more RBC-derived MPs (RMPs) in EDTA PPP (Supplemental Figure 3B). This artifact has been described previously^8^.

## Discussion

Prompted by unexpected findings in a case-control study of circulating miRNAs associated with PAD, we examined the extent of artifact caused by residual platelets in archived plasma samples. We provide clear evidence that platelet contamination confounds attempts to study extracellular miRNA in archived plasma prepared according to standard protocols, which include post-thaw attempts to remove residual platelets. The major findings of this study include: (1) miRNA levels in archived plasma prepared using standard protocols correlate with platelet count; (2) freeze/thaw of residual platelets in standard plasma results in PMP generation, release of miRNA from platelets, and contamination of the extracellular miRNA pool; (3) the effect of freeze/thaw on extracellular miRNA levels is only modestly attenuated by post-thaw removal of residual platelets; and (4) miRNA from residual platelets may account for more than 90% of the miRNA detected in inadequately processed archived plasma, although the degree of this contribution is different for each miRNA. Since archived plasma has been utilized in numerous published and ongoing clinical studies of extracellular miRNAs, our findings have widespread implications in this field.

The number of residual platelets in standard plasma preparations is influenced by sample processing, as has been shown previously^7^, or simply by differences in baseline platelet count in blood samples processed similarly, as we describe here. In standard plasma preparations, the number of residual platelets after initial centrifugation is probably related to the platelet count in whole blood. Importantly, slight differences in sample processing, such as centrifugation speed and sample volume, could result in systematic differences in the number of residual platelets and, subsequently, lead to batch effects. This is a legitimate concern, as it is not uncommon for biomarker studies to compare archived plasma samples prepared at different sites, each using different protocols and equipment.

The fact that many recent reports of circulating miRNA profiles, published in high-impact journals, give few, if any, details regarding plasma preparation suggests a lack of awareness among both investigators and reviewers that plasma processing critically impacts the measurement of extracellular miRNA. It may be commonly overlooked or not widely known that large numbers of platelets remain in standard plasma preparations of EDTA anticoagulated blood. Methods for plasma processing were reviewed in 42 extracellular miRNA-disease association studies published since 2008 (Table 1). Over two-thirds of these studies either did not remove residual platelets from plasma or do not provide enough detail to make this determination.

Platelets have a large repertoire of miRNAs^9^ and are a major source of miRNAs in plasma^10^. Studies by Cheng *et al*. and Kaudewitz *et al*. suggest that, in standard plasma preparations, the amount of intracellular miRNA in platelets is much greater than the levels found in the extracellular space^7^,^10^. Our data are in agreement with their findings. While some true extracellular miRNAs, such as the RBC-specific miR-451, are not expressed in platelets and thus are not affected by post-thaw release of intracellular platelet miRNAs, most other true extracellular miRNAs are also expressed in platelets. Therefore, studies involving archived plasma samples that were not cleared of platelets prior to storage have primarily assessed the residual platelet contamination of each sample^1^,^2^,^4^. Similar to us, Zampeteki and co-workers observed very strong correlations for most miRNAs measured in their study. Importantly, the few miRNAs that displayed low correlation (miR-486, miR-122) in their study are not appreciably expressed in platelets^2^. It would be interesting to know whether this group could identify a correlation between whole blood platelet counts and miRNA levels in their samples.

Cheng *et al*. elegantly demonstrated that plasma preparation and residual platelets can have profound effects on miRNA measurements^7^. This group tested whether the effects of residual platelets in archived standard plasma could be mitigated by post-thaw processing. They compared MP levels, platelet counts, and miRNAs in archived standard plasma before and after an additional, post-thaw centrifugation step. They concluded that additional centrifugation was effective in removing residual platelets and potential intracellular platelet miRNA contamination. However, they did not compare fresh PPP with ptPPP. As such, they did not observe, as we have here, that the majority of MPs and miRNAs detected in thawed standard plasma, even after additional centrifugation, were likely platelet MPs and miRNAs released during the freeze/thaw process.

The effects of freeze/thaw on residual platelets in standard plasma are not surprising. Platelets are well known to be activated by cooling and/or freezing, and it has been shown that platelet activation leads to a loss of membrane integrity^11^,^12^. We show that membrane integrity of residual platelets undergoing a freeze/thaw cycle was compromised, as measured by increased calcein-AM detected in the eluent of labeled platelets. We also found a dramatic increase in the number of platelet-derived MPs after freeze/thaw, accompanied by a marked increase in miRNAs known to be expressed in platelets, such as miR-21 and miR-27b. It is evident that, in archived plasma samples, freeze/thaw-induced changes in residual platelets will irreversibly contaminate the true extracellular miRNA pool with intracellular platelet miRNA. These findings are supported by the strong correlation between baseline platelet counts and plasma miRNA levels observed in our case-control study.

The possibility of normalizing miRNA data to account for residual platelet artifact seems an unlikely solution, as the bulk of miRNA measured in these samples will be platelet-derived, with a relatively small contribution from true extracellular miRNA. Furthermore, normalization would require making multiple assumptions, all of which are uncertain: 1) the freeze/thaw effect occurs to the same degree in each sample; 2) normalization can be applied to large numbers of different miRNAs that each, presumably, have a unique ratio of platelet to extracellular level at baseline; 3) all miRNAs released from platelets are equally protected from endogenous RNase activity. Given the uncertainty of these assumptions, the plausibility of identifying an effective normalization strategy for detecting differences in true extracellular miRNA abundance is low.

There was less platelet miRNA contamination observed in plasma isolated from citrate-anticoagulated blood compared to plasma from EDTA-anticoagulated blood. As a potential explanation, we routinely observed fewer residual platelets in standard preparations of citrate plasma as compared to EDTA plasma. Furthermore, the dimensions of a blood collection tube can substantially affect the centrifugal force applied to the sample and the number of residual platelets in plasma. The volume of whole blood in EDTA vacutainers is typically 10 mL, whereas citrate vacutainers used in this study were 2.7 mL. When equal volumes of EDTA and citrate-anticoagulated blood were compared, we observed comparable numbers of residual platelets, though there tended to be fewer in citrate (Supplemental Figure 3A). This phenomenon has been observed previously and may be related to increased platelet aggregation in citrate, thereby resulting in greater removal of platelets with other cells during initial plasma separation^13^.

Sodium citrate is recommended as the optimal anticoagulant for the study of MPs^14^, although a recent report suggests that acid-citrate dextrose (ACD) may be superior^15^. Based on our observations and those of others^8^, blood collection in EDTA may be associated with MP artifacts, particularly the post-collection generation of RBC MPs. Thus, we recommend collecting blood in sodium citrate or ACD when studying MPs and MP-encapsulated miRNAs. Prior to freezing samples (if necessary) or RNA isolation, plasma should be centrifuged a second time to remove residual platelets. In our hands, 1900x g for 10 minutes is sufficient to remove the majority of platelets, but not all (Supplemental Figure 1). The International Society of Thrombosis and Hemostasis (ISTH) recommends an initial centrifugation of 2,500x g for 15 minutes to prepare PPP, and an additional centrifugation of 2,500x g for 15 minutes for the preparation of platelet free plasma (PFP)^8^,^15^,^16^. We find that this protocol is effective (Supplemental Figure 2), although we again observe differences in the number of residual platelets depending on the centrifuge used and the volume of plasma undergoing centrifugation. Investigators should independently verify that their protocol effectively removes platelets.

Typically, extracellular miRNA is defined as that which circulates in MPs and exosomes, or bound to proteins (e.g. Ago2) and lipoproteins (e.g. HDL)^17^. Comprehensive study of extracellular miRNA in plasma is complicated by the fact that low speed centrifugation used to remove blood cells may also remove a substantial number of MPs^18^. We have observed that additional centrifugation of standard plasma (to remove residual platelets) also results in the loss of MPs (Supplemental Figure 1). This effect could be avoided by sorting MPs from whole blood using fluorescence activated cell sorting (FACS) technology. Thus, a complete picture of the true extracellular miRNome in blood will likely require a combination of multiple blood processing methods.

We acknowledge that the sample size used to evaluate PPP versus ptPPP were small. However, because the effect of freeze/thaw on residual platelets is so dramatic and consistent, studying additional samples is unlikely to provide any additional information.

Extracellular miRNAs are promising disease biomarkers, but sample processing has a significant influence on miRNA levels detected. Plasma samples that have been frozen prior to the removal of residual platelets—reports of which abound in the literature—will be highly contaminated by the artifactual release of intracellular platelet miRNA and may not be ideal for biomarker studies of extracellular miRNA. Care should be taken to standardize all aspects of blood collection and plasma processing, and detailed methods should be included with published findings.

## Methods

### Subjects and samples

Archived plasma samples from individuals with significant PAD (cases) and those with risk factors but no clinical PAD (controls) were identified in established repositories at Emory University. The PAD plasma samples were collected from participants in the Granulocyte-Macrophage Stimulating Factor in the Treatment of Peripheral Arterial Disease (GPAD-2) clinical trial^19^ and the control samples were collected from participants in the Emory/Georgia Tech Center for Discovery Health and Well Being (CDHWB) registry. Twenty-nine cases and 31 controls were matched for cardiovascular risk factors using a propensity score composed of age, gender, race, diabetes, hyperlipidemia, smoking, HDL, LDL, systolic and diastolic blood pressure, and serum creatinine (Supplemental Table 1). For both cohorts, fasting blood had been collected in ETDA-anticoagulated vacutainers and centrifuged at 3,000 RPM for 10 minutes to sediment cells. Plasma was isolated and immediately stored at −80 °C without further processing. The Emory University Internal Review Board (IRB) approved all studies. Informed consent was obtained from all human subjects and all methods were performed in accordance with the relevant guidelines and regulations.

### RNA isolation

Plasma samples were thawed at 37 °C and centrifuged at 1,900x g for 10 minutes to remove residual cells and debris^7^. For each sample 500 μl of Qiazol (Qiagen) was added to 100 μl of plasma. Synthetic exogenous oligonucleotides were then added, as recommended by the manufacturer (Exiqon or Qiagen). For samples that underwent complete profiling in the discovery phase, synthetic UniSp2, UniSp4, and UniSp5 were added prior to RNA isolation to monitor for isolation efficiency, and UniSp6 and Cel-39 were added prior to reverse transcription (RT) to monitor RT-PCR efficiency (Exiqon). For samples studied in the validation phase, 5 μl of *C. elegans* miR-39 spike-in control obtained from Qiagen was added to monitor for technical variation. RNA was isolated using the miRNeasy Mini Kit according to the manufacturer’s protocol (Qiagen).

### miRNA profiling array

In the discovery phase, plasma miRNA profiling was carried out using Exiqon’s Serum/Plasma Focus microRNA PCR Panels. 4 μl of RNA eluted in water was utilized for RT, and PCRs were carried out according to the manufacturer’s protocol (Exiqon) on a StepOne Real-Time PCR System (Thermofisher). Data was analyzed using the GeneEx qPCR software package (Exiqon). Raw Ct values were corrected for inter-plate variation, samples were normalized to the exogenous spike in miRNAs, converted to relative expression values (2^−ΔCt^), and log transformed for statistical analysis.

### RT-qPCR

The miScript II RT Kit (Qiagen) and miScript SYBR Green PCR Kit (Qiagen) were used for reverse transcription and qPCR, respectively, in the validation phase. RTs were performed in 10 μl reaction volumes using 6 μl of RNA eluted in water as template. PCRs were performed using commercially available primers available through Qiagen on a StepOne Real-Time PCR System (Thermofisher).

### Flow cytometry

The following fluorescent labeled primary antibodies were incubated with 14 μl plasma (20 minutes, room temperature): 0.125 μg/ml f.c. PE/Cy5 anti-CD41 (Biolegend cat. 303708) or isotype control (Biolegend cat. 400115); 0.25 μg/ml f.c. PE/Cy7 anti-CD45 (eBioscience cat. 25-0459-41) or isotype control (eBioscience cat. 25-4714-80); 0.25 μg/ml f.c. APC anti-CD146 (BioLegend cat. 342011) or isotype control (BioLegend cat. 400221); 0.125 μg/ml f.c. PE anti-CD235a (eBioscience cat. 12-9987-80) or isotype control (eBioscience cat. 12-4732-41). The non-specific fluorescent dye, Calcein-AM, was added to PPP at a final concentration of 10 μM and incubated (20 minutes, 37 °C). Stained plasma was diluted in PBS prior to analysis on a BD LSRII or a BD FACS Aria II flow cytometer. Appropriate compensations were performed for multicolor assays.

### Effect of freeze/thaw on MPs and miRNA detected in PPP

Blood was collected into EDTA and citrate vacutainers from the antecubital veins of six healthy volunteers. The first 5 mL of blood collected was discarded, and a fresh vacutainer was subsequently filled. Whole blood was processed immediately by centrifugation at 3,000 RPM (1,800× g) for 10 minutes in an Eppendorf 5810 R Centrifuge at 4 °C. Plasma was removed with caution to avoid disrupting the buffy coat, and was labeled as “standard plasma”. For each sample, half of the standard plasma was frozen at −80 °C (“frozen standard plasma”) then thawed at 37 °C for further analysis. Aliquots of standard plasma that did not undergo freeze/thaw (“fresh standard plasma”) and freeze/thaw standard plasma underwent a second centrifugation at 1,900x g for 10 minutes to generate PPP as previously recommended^7^. The second centrifugation was done in 1.5 mL Eppendorf tubes using an Eppendorf 5430 R centrifuge. MPs were measured by flow cytometry and miRNA by RT-qPCR as described above.

### Platelet membrane integrity assay

Standard plasma was obtained from healthy volunteers as described above and residual platelets were pelleted with a second centrifugation at 1,900x g for 10 minutes. The supernatant was removed and the residual platelet pellet was re-suspended in PBS. Calcein-AM was added to the resuspended pellets at a concentration of 10 uM to fluorescently label residual platelets^20^. Each sample was then split into two aliquots; one remained at room temperature while the other underwent a single freeze/thaw cycle at −80 °C. After rapid thawing of the frozen aliquots, all samples were centrifuged at 16,100x g for 20 minutes to pellet cells and MPs. 15 μl of supernatant was removed after centrifugation, diluted in a total volume of 200 ul of PBS, and fluorescence intensity (excitation = 488 nm, emission- = 520 nm) was measured in triplicate on a microplate reader.

### Measurement of miRNA in MPs and vesicle-free plasma after freeze/thaw

Standard plasma was collected from healthy volunteers and split into two aliquots: one underwent a single freeze/thaw cycle (frozen standard plasma) while the other remained at room temperature (fresh standard plasma). Aliquots (200 μl) of frozen standard plasma and fresh standard plasma each underwent sequential centrifugation to pellet residual cells (1,900x g, 10 minutes), MPs (16,000x g, 20 minutes), or exosomes (100,000x g, 90 minutes). RNA was isolated from each fraction and miRNA levels were assessed as described above.

## Statistical Analysis

Baseline characteristics of participants (GPAD and CDHWB) in the case-control study are presented as mean and standard deviation for continuous variables and percent for categorical variables (Supplemental Table 1). Differences between groups were assessed using a Mann-Whitney U test for continuous variables and a Fisher’s exact test for categorical variables. To identify miRNAs differentially expressed between the control and PAD groups, an independent t-test was performed and p-values were adjusted for multiple testing with a false discovery rate method (FDR).

To identify endogenous control miRNA from the discovery dataset as an alternative normalization strategy, all miRNAs with Ct < 34 were normalized to the global mean, log transformed, and ranked according to coefficient of variation (CV)^21^. miR-425-5p was selected as it had among the lowest CV, was expressed in all samples at a relatively high amount, has been suggested by others to be a suitable endogenous control^22^,^23^, and has no known association with CVD. For all data presented, plasma miRNA levels were normalized as 2^−(Ct target miRNA – Ct Cel-39)^. Because RNA isolation and reverse transcription were volume based, Ct values are a reflection of miRNA concentration in each sample. Pearson correlation coefficients and the statistical significance were calculated for all miRNA pairs using Prism Software (Graphpad).

## Additional Information

**How to cite this article**: Mitchell, A. J. *et al*. Platelets confound the measurement of extracellular miRNA in archived plasma. *Sci. Rep.* **6**, 32651; doi: 10.1038/srep32651 (2016).

## Supplementary Material

Supplementary Information

## Figures and Tables

**Table 1:** A review of plasma preparation protocols in extracellular miRNA-disease association studies.

Study	Disease	AC	Freeze before platelet removal	Whole blood cent. speed	Time (min)	2^nd^ cent. speed	Time (min)	Yr.	PMID	Journal	
Wong *et al*.	HFpEF	EDTA	Yes	3,488x g	10	No		2015	25619197	Eur J Heart Fail	B
Aherne *et al*.	Colorectal cancer	EDTA	Yes	1,000x g	10	No		2015	25924769	BMC Cancer	C
Melman *et al*.	Response to CRT	?	?	?	?	?	?	2015	25995320	Circulation	C
Ameling *et al*.	Associations with age, BMI, sex	EDTA	?	?	?	?	?	2015	26462558	BMC Med Genomics	C
Jaguszewski *et al*.	Takotsubo cardiomyopathy	EDTA	Yes	1,300x g	15	No		2014	24046434	Eur Heart J.	C
Xiao *et al*.	Acute graft-versus-host disease	?	No	2,000 RPM	10	12,000 RPM	3	2014	24041574	Blood	A
Garcia *et al*.	Regression of LVH	EDTA	Yes	1,389x g	30	No		2014	23948643	JAHA	C
Roncarati *et al*.	Hypertrophic cardiomyopathy	EDTA	Yes	1,100x g	20	No		2014	24161319	JACC	C
Zanutto *et al*.	Colorectal cancer	EDTA	Yes	3,000x g	10	1,000x g	5	2014	24423916	Br J Cancer	C
Huang *et al*.	Myocardial infarc	EDTA	No	1,600x g	10	16,000x g	10	2014	24627568	Circ. CV Genet	A
Morely-Smith *et al*.	Response to LVAD	?	?	?	?	?	?	2014	24961598	Eur J Heart Fail	C
Ward *et al*.	Hepatotoxicity or ischemic hepatitis	EDTA	Yes	1,500x g	20	No		2014	25092309	PNAS	C
Pangxiang *et al*.	Intracranial aneurysm	EDTA	No	1,600x g	10	13,000x g	10	2014	25249297	JAHA	A
Jansen *et al*.	Cardiovascular events	Citrate	No	1,500x g	15	13,000x g	2	2014	25349183	JAHA	A
de Boer *et al*.	Aspirin effect on miR-126	Citrate	Yes	2,700x g	15	No		2013	23386708	Eur Heart J.	B
Dawson *et al*.	Atrial fibrillation	EDTA	Yes	4,000 RPM	20	No		2013	23459615	Circulation	B
Williams *et al*.	Comprehensive sequencing	EDTA	No	500x g	5	16,060x g	5	2013	23440203	PNAS	A
Ellis *et al*.	Heart failure	EDTA	Yes	?	?	No		2013	23696613	Eur J Heart Fail	C
Ferrajoli *et al*.	B-cell lymphocytosis and B chronic lymphocytic leukemia	Citrate	?	?	?	?	?	2013	23821659	Blood	C
Lee *et al*.	Recurrent papillary thyroid cancer	EDTA	Yes	800x g	15	No		2013	24301304	Cancer	C
Zampetaki *et al*.	Myocardial Infarction	?	?	?	?	?	?	2012	22813605	JACC	C
Kin *et al*.	AAA	?	?	?	?	?	?	2012	23316282	JAHA	C
Moussat *et al*.	B-cell chronic lymphocytic leukemia	EDTA	No	1,000x g	10	10,000x g	20	2011	21460253	PNAS	A
Boeri *et al*.	Lung cancer	EDTA	No	1,258x g	?	1,258x g	?	2011	21300873	PNAS	A
Zuo *et al*.	Myelodysplastic syndrome	?	?	?	?	?	?	2011	21602527	Blood	C
Li *et al*.	Hypertension	EDTA	?	1,000x g	10	No		2011	21690488	Circulation	C
Komatsu *et al*.	Esophageal cancer	?	No	1,500 RPM	30	3,000 RPM	5 min	2011	21673684	Br J Cancer	C
Morimura *et al*.	Pancreatic cancer	Heparin	No	1,500 RMP	30	3,000 RPM	5 min	2011	22045190	Br J Cancer	C
De Rosa *et al*.	Transcoronary grandient	?	?	?	?	?	?	2011	21969012	Circulation	C
Zile *et al*.	Left ventricular remodeling	?	?	?	?	?	?	2011	21956146	Circ. CV Genet	C
Wang *et al*.	Acute myocardial infarction	EDTA	No	820x g	10	16,000x g	10	2010	20159880	Eur Heart J.	A
Tijsen *et al*.	Heart failure	Citrate	Yes	1,550x g	10	No		2010	20185794	Circ Res	B
Ho *et al*.	Pancreatic cancer	?	Yes	3000 RPM	10	13,000x g	10	2010	20360935	Transl Oncol.	C
Tsujiura *et al*.	Gastric cancer	?	?	?	?	?	?	2010	20234369	Br J Cancer	C
Zampetaki *et al*.	Tyoe 2 diabetes	?	Yes	?	?	?	?	2010	20651284	Circ Res	C
Fichlscherer *et al*.	Coronary artery disease	EDTA	?	?	?	?	?	2010	20595655	Circ Res	C
D’Alessandra *et al*.	Myocardial infarction	EDTA	No	1,500x g	15	14,000x g	15	2010	20534597	Eur Heart J.	A
Maarten *et al*.	Myocardial damage	Citrate EDTA	Yes	?	?	?	?	2010	20921333	Circ CV Genet.	C
Wang *et al*.	Drug induced liver injury	?	?	?	?	?	?	2009	19246379	PNAS	C
Tanaka *et al*.	Acute leukemia	?	no	1,600x g	15	No		2009	19440243	PLoS One	C
Mitchell PS *et al*.	Prostate cancer	EDTA	yes	1,200x g	10	No		2008	18663219	PNAS	C
Ng *et al*.	Colorectal cancer	EDTA	no	1,600x g	10	16,0000x g	10	2008	19201770	Gut	A

AC = anticoagulant; A = likely removed platelets; B = unclear whether platelets were removed; C = unlikely platelets were removed or not enough detail in methods to determine.
