# Supplementary material for: Platelets confound the measurement of extracellular miRNA in archived plasma
Source: Sci Rep. 2016 Sep 13;6:32651. doi: 10.1038/srep32651 (PMC5020735; doi:10.1038/srep32651)

Supplementary material

Platelets confound the measurement of extracellular miRNA in archived plasma

Adam J. Mitchell MD[1]*; Warren D. Gray PhD[1]*; Salim S. Hayek MD[1]; Yi-An Ko PhD[3]; Sheena Thomas[1]; Kim Rooney[1;] Mosaab Awad MD[1]; John D. Roback MD, PhD[4]; Arshed Quyyumi MD[1]; Charles D. Searles MD[1,2*]

[1]Division of Cardiology, Department of Medicine, Emory University, Atlanta, GA
[2]Section of Cardiology, Atlanta VA Medical Center, Decatur, GA
[3]Department of Biostatistics, Rollins School of Public Health, Emory University, Atlanta, GA
[4]Department of Pathology, Emory University, Atlanta, GA

*AJM and WDG are co-first authors

Supplemental Table 1. Baseline characteristics of the case-control cohort

Variable	Controls (N=31)	PAD (N=29)	P-value
Age, years	58 ± 10	62 ± 8	0.10
Male, %	25 (81)	29 (100)	0.02
Black, %	6 (19)	8 (28)	0.55
DM, %	6 (19)	12 (41)	0.09
Smoking, %	5 (16)	8 (28)	0.35
Hypertension, %	23 (71)	22 (76)	1.00
Hyperlipidemia, %	18 (58)	26 (90)	0.01
Weight, kg	87 ± 20	88 ± 16	0.70
BMI, kg/m^2	29 ± 5	28 ± 5	0.73
SBP, mm Hg	128 ± 14	128 ± 17	0.87
DBP, mm Hg	81 ± 10	74 ± 11	0.04
LDL (mg/dL)	95 ± 28	93 ± 33	0.71
HDL (mg/dL)	56 ± 17	47 ± 15	0.02
Glucose (mg/dL)	94 ± 8	102 ± 29	0.90
Creatinine (mg/dL)	0.9 ± 0.2	1.1 ± 0.5	0.02
Ankle-brachial Index	-	0.59 ± 0.03	
Aspirin	17 (55)	21 (72)	0.18
Plavix	0 (0)	6 (21)	0.01
Statin	18 (58)	22 (76)	0.09
Beta blocker	3 (10)	18 (62)	<0.0001
ACE/ARB	14 (45)	12 (41)	0.60
Calcium channel blocker	3 (10)	8 (28)	0.19
Cilastazol	0 (0)	8 (28)	0.002

Values are mean ± standard deviation or n (%) where appropriate.

Supplemental Figure 1.

Effect of centrifugation speed on the removal of residual platelets from standard plasma. Standard plasma was prepared and aliquots underwent a second centrifugation for 10 minutes at 400, 600, 1000, 1500, and 1900 x g to assess remove of residual platelets. Calcein-AM was added to the resultant supernatant of each spin, which was then analyzed by flow cytometry. The Y-axis is side scatter (SSC) and the X-axis is FITC (calcein intensity). Calcein-AM intensity can effectively distinguish platelets (high intensity, black) from MPs (low intensity, red). Centrifugation at 1900 x g effectively removed >99.99% of platelets present in standard plasma, though more than 80% of the MP population is also lost.

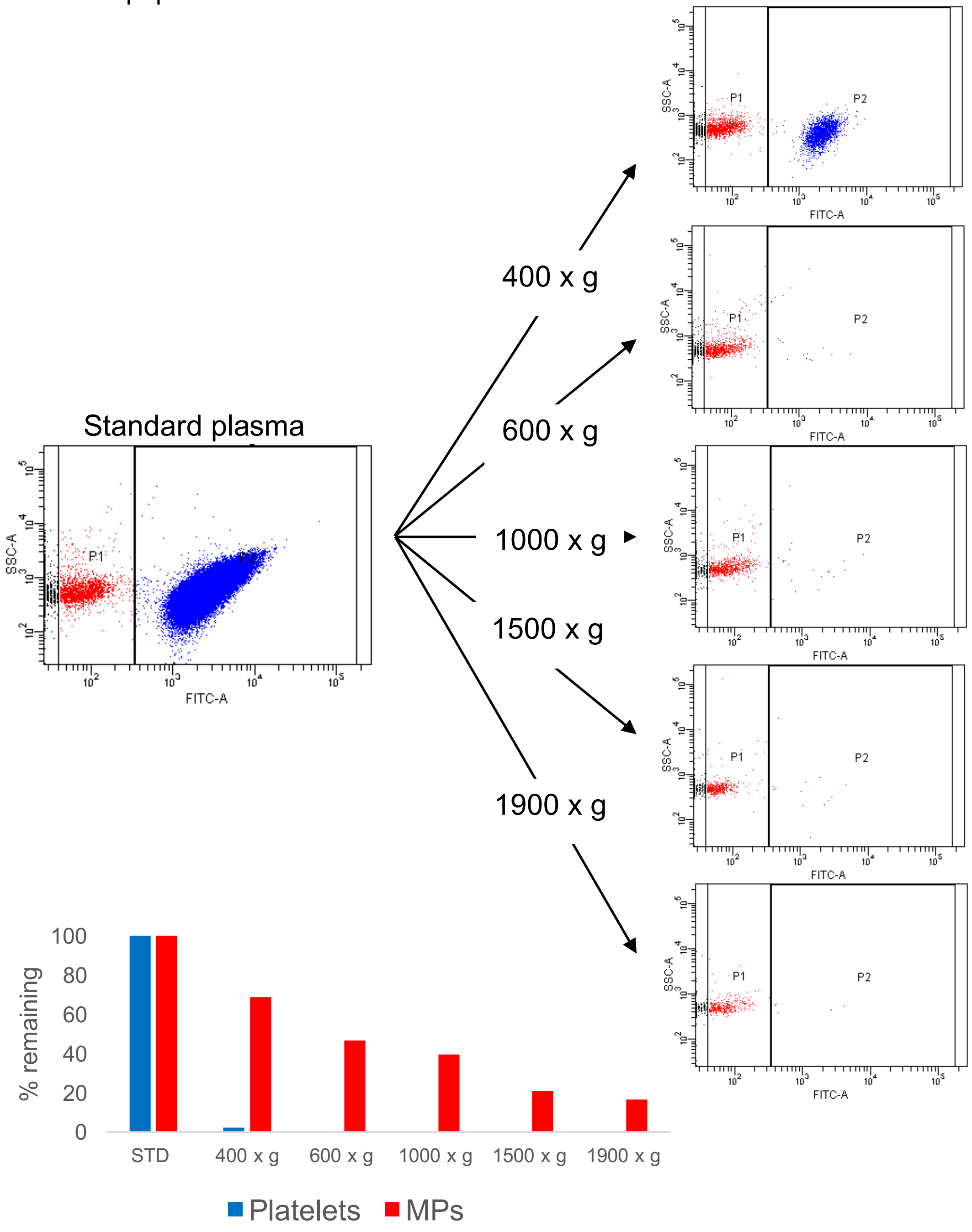

Supplemental Figure 2.

Flow cytometry gating strategy to distinguish platelets and MPs.

Citrate anticoagulated blood was processed according to the International Society of Thrombosis and Hemostasis (ISTH) guidelines to generate platelet poor plasma (PPP) and (PFP). 50μl of each sample was left unstained, or incubated for 20 minutes with the appropriate isotype control, 10μM calcein-AM, anti-CD41-PE-Cy5, or double stained (calcein, CD41) and analyzed on a BD FACS Aria II flow cytometer. (A) The X-axis reports FITC (calcein intensity) and the Y-axis reports CD41, a platelet antigen. Q1 was defined as platelet MPs (CD41+, low calcein intensity), Q2 as platelets (CD41+, high calcein intensity), and Q3 as non-platelet derived MPs. (B) A second centrifugation to generate PFP effectively removes all platelets (Q2).

A PPP prepared according to ISTH guidelines (2,500 x g, 15 minutes)

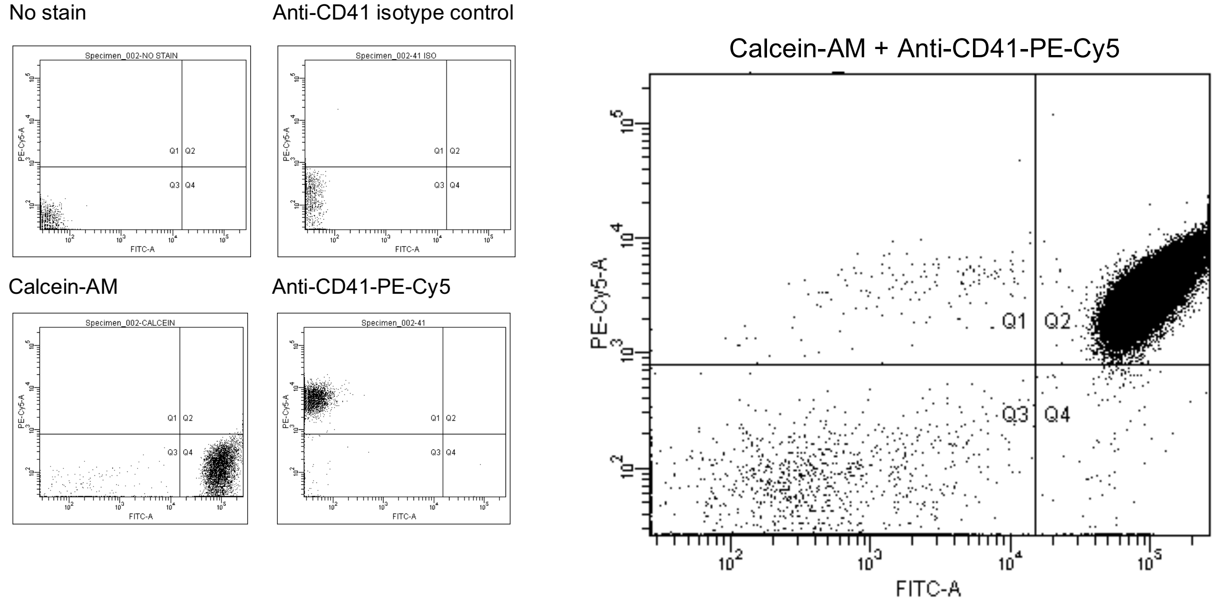

B PFP prepared according to ISTH guidelines (additional 2,500 x g, 15 minutes)

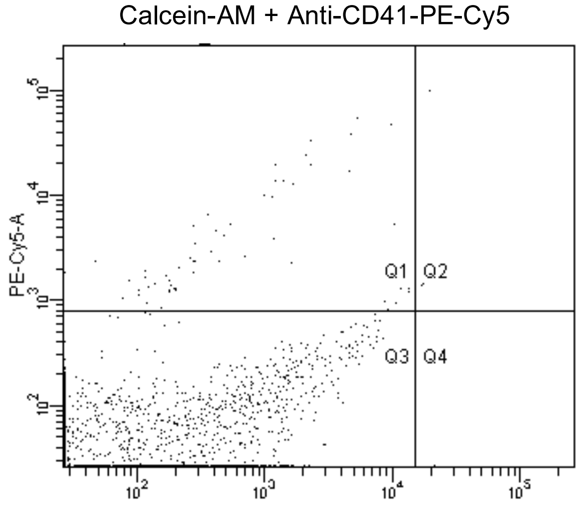

Supplemental Figure 3.

Increased number of residual platelets and RBC MPs in EDTA as compared to citrate-anticoagulated plasma. (A) Whole blood was drawn from a single healthy volunteer into either EDTA or citrate vacutainers. After mixing of blood with anticoagulant, equal volumes (3mL) were transferred to a 15mL tube and centrifuged at 3000 RPM (1800 x g) to prepare standard plasma. Standard plasma was stained with calcein. There were notably fewer events in both the platelet gate (green) and the MP gate (red) in citrate plasma (events/minute, equal flow rate). (B) EDTA and citrate platelet poor plasma prepared in parallel from the same donor was stained with anti-CD235a to quantify RMPs (events/minutes, equal flow rate). Gate P2 (red) was defined as CD235a+. We routinely observe an increased number of CD235a+ MPs (RMPs) in EDTA plasma than in citrate, an artifact that had been described before.

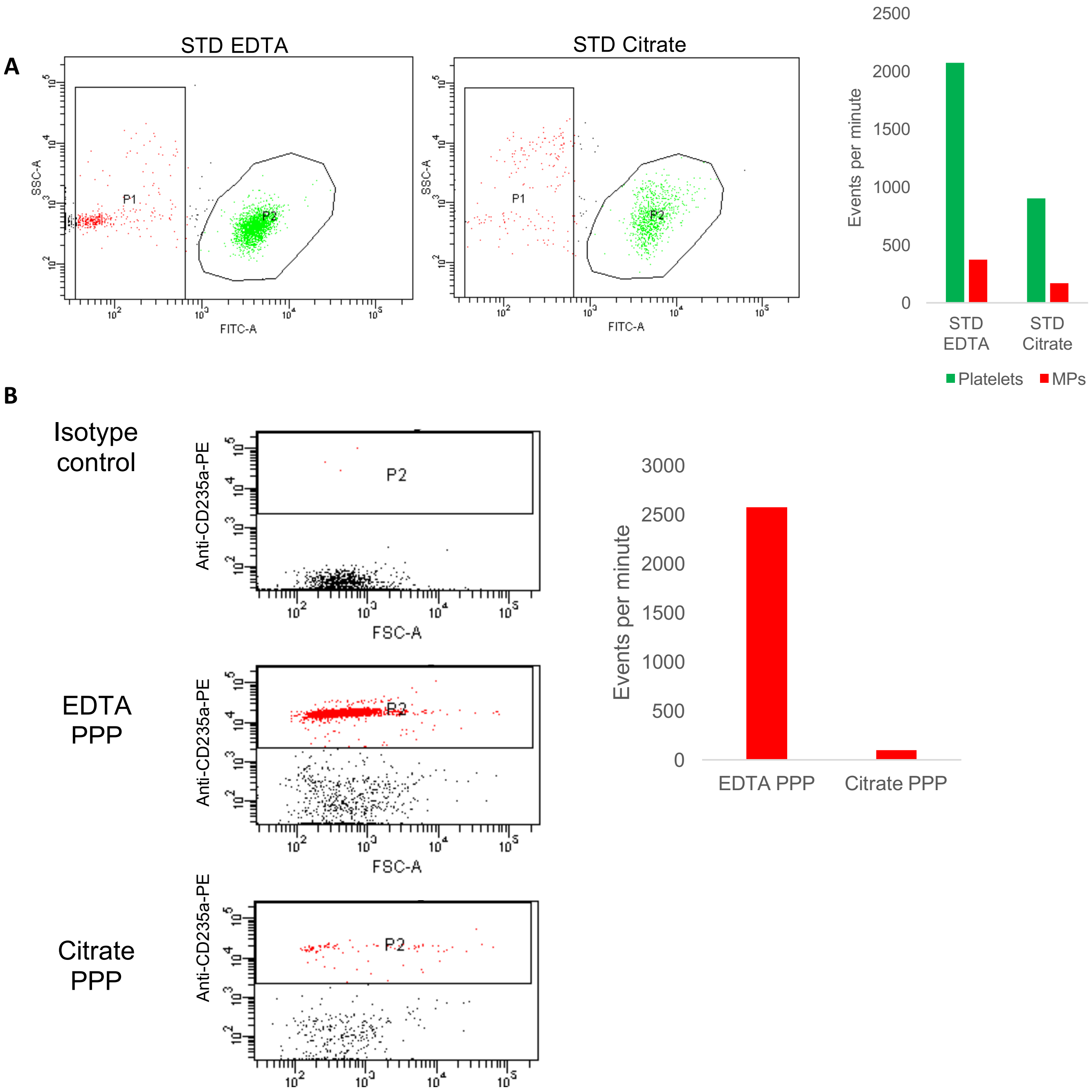

Supplement: Supplementary Information [file srep32651-s1.pdf]
